# Group Effects on Individual Attitudes Toward Social Responsibility

**DOI:** 10.1007/s10551-016-3106-x

**Published:** 2016-03-15

**Authors:** Davide Secchi, Hong T. M. Bui

**Affiliations:** 1 COMAC Research Cluster, Centre for Human Interactivity (CHI), Department of Language and Communication, University of Southern Denmark, Sdr. Stationvej 28, 4200 Slagelse, Denmark; 2 Southampton Management School, University of Southampton, Highfield, Southampton, SO17 1BJ UK; 3 VNUK Insitute, University of Danang, Danang, Vietnam

**Keywords:** Individual social responsibility, Absolute and relative social responsibility, Group dynamic, Socialization, Attitudes toward social responsibility

## Abstract

This study uses a quasi-experimental design to investigate what happens to individual socially responsible attitudes when they are exposed to group dynamics. Findings show that group engagement increases individual attitudes toward social responsibility. We also found that individuals with *low* attitudes toward social responsibility are *more* likely to change their opinions when group members show more positive attitudes toward social responsibility. Conversely, individuals with *high* attitudes do not change much, independent of group characteristics. To better analyze the effect of group dynamics, the study proposes to split social responsibility into *relative* and *absolute* components. Findings show that *relative* social responsibility is correlated with but different from *absolute* social responsibility although the latter is more susceptible than the former to group dynamics.

Social responsibility (SR) has been traditionally associated with different perspectives, such as the traditional theory of the firm (Lim and Phillips 2008; McWilliams and Siegel 2011; Ormiston and Wong 2013), stakeholders (Freeman and Liedtka 1991; Hillenbrand et al. 2011), stewardship (Van Dierendonck 2011), sustainability (Gully et al. 2013; Van Marrewijk 2003), corporate citizenship (Glavas and Piderit 2009; Küskü and Zarkada-Fraser 2004), recruitment (Greening and Turban 2000; Jones et al. 2014), job satisfaction (Bauman and Skitka 2012), and many more. The majority of these perspectives address the so-called corporate or organizational level of analysis (Bondy and Starkey 2012; Carroll and Shabana 2010). Traditionally, the study of SR has been concerned with the relations between businesses and the external environment, as most of the reviews of the literature seem to suggest (Carroll and Shabana 2010; Garriga and Melé 2004; Secchi 2007). In other words, it studies the position and behavior of the organization as a whole. In contrast, socially responsible perceptions and behavior of agents within organizations featured less prominently in the literature, as already highlighted by Aguinis and Glavas (2012) and earlier by Windsor (2001). Although still less studied than the former, research on this latter aspect of SR is growing (e.g., Aguilera et al. 2007; Rupp 2011; Rupp et al. 2013) and can be referred to as individual SR (ISR). This stream of research is concerned with the way individuals perceive organizational social responsibility (e.g., Greening and Turban 2000; Garcia de los Salmones et al. 2005) and with what characterizes them as socially responsible persons (e.g., Crilly et al. 2008; Secchi 2009).

In a business environment, SR is often considered, implemented, and processed into procedures and routines, or even incorporated into the company’s strategy by individuals. It is the company’s executives, managers, and employees who make decisions on various aspects of SR (Aguilera et al. 2007; Aguinis and Glavas 2012). A further look at the topic reveals that decisions on SR might also be made collectively by groups/teams (Cappel and Windsor 2000; Rupp et al. 2006; hereafter the words ‘group’ and ‘team’ are used interchangeably). In addition, group or team work is increasingly becoming part of regular business and organizational structures (Colquitt et al. 2002; Cropanzano and Schminke 2001). Although groups are key in everyday organizational life, the relationship between SR and group dynamics is yet to be explored. For example, how are individual attitudes toward SR affected by exposure to group activities? Is SR thinking, behavior, and attitude dependent upon the group? Answering these questions will help develop a theory of how group dynamics affect individual SR, and assist further understanding of the role of groups in relation to individual perceptions and behaviors of SR. For the purpose of this study we maintain that ISR reflects a disposition toward making decisions based on a conscious understanding of the consequences that business operations have on both stakeholders and wider society as a whole. This article is concerned with attitudes toward SR, defined consistently with the literature (Gough et al. 1952; Rupp 2011; Sully de Luque et al. 2008) and located under the umbrella of *relational* theories of SR (Rupp and Mallory 2015; Secchi 2007).

Therefore, moving from ‘gap-spotting’ to ‘path setting’ (Alvesson and Sandberg 2011; 2013), we focus on (1) how individual attitudes change when one is exposed to group activities; (2) what the role of the group is when perceptions of SR are considered; and (3) how individuals are affected by group members in formulating opinions on SR. Using a quasi-experimental design we collected data in two waves, one that took place in 2011, the other in 2012.

## Theory and Hypotheses Development

Many studies on SR tend to focus on both ethics and social responsibility together (Carroll and Shabana 2010; Kolodinsky et al. 2010; Singhapakdi et al. 1996). The rationale for this is twofold. SR has firstly emerged from and has been traditionally attached to studies on business ethics and/or business and society (Garriga and Melé 2004; Secchi 2007). For this historical reason, most of the previous studies on individuals do not attempt to isolate ethical from SR perspectives. The other reason concerns the perception that socially irresponsible business practices are somehow understood and studied on moral grounds. This second element derives from the first although it deals more with how scholars frame SR, up to the point where some explicitly refer to ‘normative’ elements in particular branches of CSR (e.g., in international business—see De George 2000; in normative stakeholder approaches—see Donaldson and Preston 1995). If these two perspectives are the same, then there may be no need to separate them. However, if SR and business ethics describe different but intertwined aspects, we should clearly separate and distinguish between the two. In the following, we try to define individual SR as separate although not completely independent from individual ethical attitudes.

**Fig. 1:**
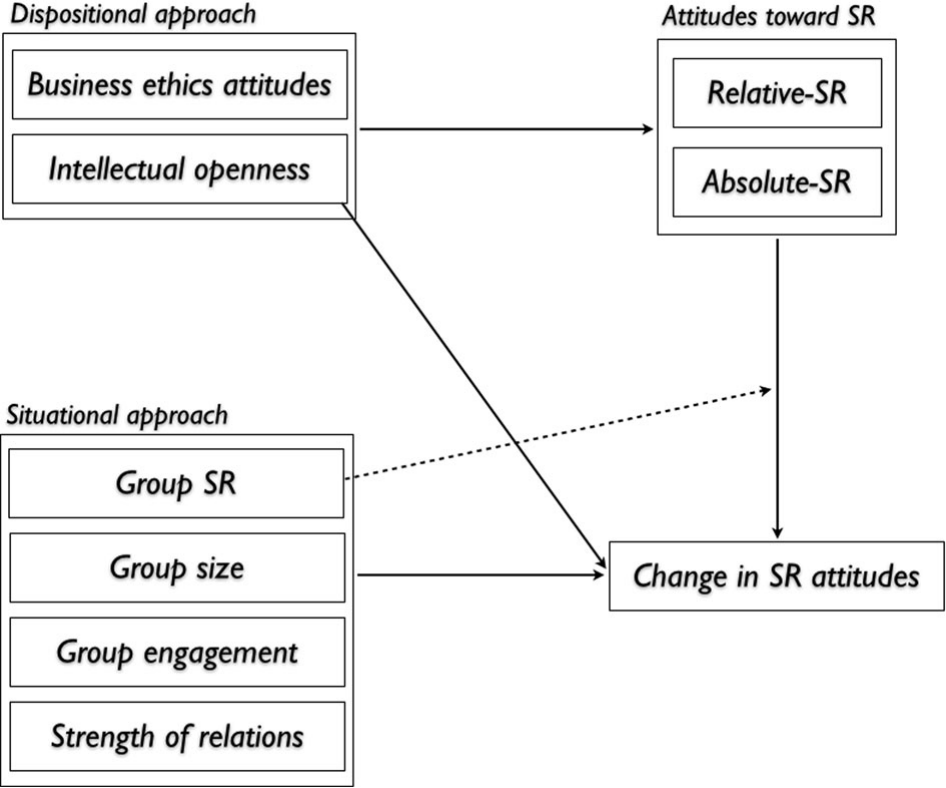
A theoretical framework for the study of group effects on individual attitudes toward SR

To date, few attempts have been made to identify the different components or antecedents of individual SR (Mudrack 2007; Muller and Kolk 2010; Rupp et al. 2006). Although group socialization mechanisms have been widely studied (Levine and Moreland 1994; Moreland and Levine 2002), and SR research has increasingly covered some aspects at the individual level of analysis (Greening and Gray 1994; Muller and Kolk 2010; Muthuri et al. 2009; Ramus and Steger 2000), we are not aware of many studies that address how group dynamics potentially affect SR. The only exception is that of Mathisen et al. (2013) which investigates how female directors of corporate boards of directors experience boardroom dynamics. Therefore, setting a theoretical framework for research on individual SR and group dynamics is not only ‘gap-spotting’ but also ‘path-setting’ (Alvesson and Sandberg 2011; 2013). In doing so, we explore the different components of the SR construct, and demonstrate how they relate to group dynamics and interactions. Figure 1 succinctly summarizes the key elements of the model we are testing and is explained in the following pages.

### Attitudes Toward Social Responsibility

Attitudes toward SR are usually referred to as individual perceptions of general corporate policies to implement social responsibility (or CSR). For example, some authors classify them into four types; i.e., economic, legal, ethical, and philanthropic/discretional (Angelidis and Ibrahim 2004; Ibrahim et al. 2008). Some other authors (Rodrigo and Arenas 2008) isolate two types of employee attitudes toward CSR programs: attitudes toward the organization and attitudes toward society. In their 2008 study, Rodrigo and Arenas reveal an attitudinal typology: the *committed* employee, the *indifferent* employee, and the *dissident* employee.

Although this approach is extremely valuable in defining how individual employees perceive their workplace (Gully et al. 2013; Rupp et al. 2013) and provides a practical view on SR, it ignores a more normative or value-based perspective to some extent. Some scholars have long argued for normative elements of certain theories of CSR, such as the stakeholder approach (Donaldson and Preston 1995; Bowie 1991). Others have tried to integrate SR with more ethical (Ormiston and Wong 2013; Singhapakdi et al. 1996) or value-based (Jones et al. 2014) aspects, supporting the idea that there is something more than just a practical aspect to SR.

Before exploring the proposal for a new classification of attitudes toward SR (see below), there is a need to understand what the relation between SR (or ISR), values/principles, and behavior is. On the one hand, ISR relates to other mental frames and states such as cognition (Crilly et al. 2008; Secchi 2009), creativity (Glavas and Piderit 2009), personality (Berkowitz and Lutterman 1968), ethics (Trevino 1986), or justice (Aguilera et al. 2007). From these studies, SR is defined as something that either affects or is derived from a particular set of individual dispositions. In fact, some scholars (in particular the work of Berkowitz and colleagues: Berkowitz and Connor 1966; Berkowitz and Daniels 1964; Berkowitz and Lutterman 1968) have attempted to define general traits of the socially responsible individual, as if there is a general attitude toward SR that is not related to a specific condition, situation, or environmental constraint. From this perspective, SR can be framed as a mindset that individuals have toward the role (and function) of businesses in society.

On the other hand, SR may eventually have an impact on organizational behavior as it has been related to commitment and identification (Kim et al. 2010), involvement (Jones et al. 2014), job attractiveness (Turban and Greening 1997), satisfaction (Witt and Silver 1994), and person-organization fit (Gully et al. 2013), among other organizational variables (Secchi 2012). In the literature, we could find little evidence that ISR (and its variations) can be interpreted as “behavior,” although there is some evidence that it is likely to *affect* behavior. Under these assumptions, SR can be framed more appropriately as an attitude, i.e., a mental disposition that enables individual (employees, managers, job applicants, and other stakeholders) actions in line with their perception of the organization’s overall social responsibility. In other words, the mix of prosocial behaviors that emerge from ISR is in line with one’s understanding of the company’s role in society. This leads, for example, to job applicants favoring a socially responsible company over a non-socially responsible company (Turban and Greening 1997) or to an increase of employee identification with the organization (Carmeli et al. 2007). In this perspective, ISR can act as a bridge between the perception of the company’s policies and individual behavior. In a different but similar conceptualization of ISR, research shows that proactive corporate citizenship positively affects an employee’s commitment to the organization (Maignan et al. 1999). Similar results can be found in relation to organizational citizenship behavior and altruism (Jones 2010; Lin et al. 2010). These aspects are clearly related to behavior while the others mentioned above (e.g., organizational attractiveness, identification) lie more at the psycho-cognitive level. Nevertheless, the perception of SR (as it relates to a given company) has been shown to significantly affect several aspects of one’s thinking and behavior.

Too little is known on how individuals react to SR, but the evidence we collected seems to highlight that there are two sides to the matter. One is the abstract idea (the values or principles) that an individual has of the role and function of businesses in society; the other is a more pragmatic understanding of the impact of a particular organization on one’s thinking and behavior (we may call this *orientation*). We may also hypothesize a positive match between the first and the second, in the same way norms affect one’s intention to behave (e.g., Ajzen 2005).

In order to capture these nuances of how individuals perceive SR, we suggest that one may have more general or abstract ideas that may or may not necessarily translate into practice. There should be some consistency between the ‘general’ (*absolute*) and the ‘particular’ or ‘applied’ (*relative*), as individuals tend to think of themselves as rational beings (Kunda 1999). In other words, we expect a consistent and positive relation between one’s ethical or value-based position and their attitude toward a more ‘absolute’ perception of SR. However, when individuals deal with practical matters, they may change and adapt their judgment (Payne et al. 1993; Weick and Roberts 1993) more than when they deal with abstractions and generalizations (Ajzen 2005; Magnani 2007). Similarly to what has been argued for ethics (Shawver and Sennetti 2009), we distinguish between *relative*-*SR*, that deals with *ad hoc* situations, problems, and issues, and a more abstract and general *absolute*-*SR*, that tends to refer to what one believes all businesses should/must do. There may be some ground to advance a direction in the hypothesized relation between these two aspects of social responsibility. If we hypothesize that reasoning about practical matters or actually doing something is derived from more abstract ideas, we may assume that individuals ‘infer’ their behavior from higher-level reasoning. This may be represented as some sort of deductive reasoning. Instead, if we hypothesize that the opposite is the case, and that practical matters help form more general ideas, then we seem to lean toward inductive reasoning. While deduction is mostly advocated by theories of rational choice (e.g., March 1994), induction is more likely to appear when individuals apply bounded rationality (e.g., Secchi 2011). Whether to lean toward the one or the other appears to be, given these two approaches, a matter of which theoretical framework we choose for our study; however, it is likely that these two mechanisms feed on each other. This is more connected to what some have called ‘abductive reasoning’ (Magnani 2009) or *abduction* (Pierce 1955), i.e., the generation of explanatory hypotheses based on some empirical evidence. Abduction needs both a certain level of abstraction and experiential reasoning; it is hypothesizing that, for example, publishing an annual social report generates more profits because the company has started to do so since that practice began. There may well be other explanations, though, and this makes this type of abduction particularly “risky” (Thagard 2012). This is the point we are trying to make. In abductive reasoning there is a mix of both practical exposure (or manipulation; see Magnani 2007) and abstractions, so that the hypothesis is generated by the interaction between the two rather than by a specific causal mechanism. Going back to the example above, one’s value may be in line with transparent accountability which leads a company to publish an annual social report. In this example, there is an alignment between one’s values and what also seem to be the company’s values: an increase in profits may be a reflection of the increased role of stakeholders who also value the improved transparency and disclosure in the company’s practices (i.e., the annual social report). There are practical elements (e.g., profits, annual report) that merge with and reinforce existing values, although these practical elements are also affected (or biased, we may posit) by the original values. However, a full exploration of these relations does not fall within the main purpose of the current study. Hence, the current study focuses on whether there is a positive correlation between the two concepts, leaving further and more informed hypotheses to future studies:

#### **H1a**

There is a more practical attitude toward SR, or *relative*-*SR*, which is correlated with but different from a more abstract SR attitude, or *absolute*-*SR*.

When working in groups, individuals tend to adapt and update their cognition (Cannon-Bowers and Salas 2001) more often than when they deal with a problem in isolation. This suggests that practical implications of a particular issue dealt with by group work helps members to consider it from various perspectives. Those who maintain a strong and abstract understanding of the issue may be less likely to change their mind. Therefore, we argue that there tends to be a stronger prevalence of and adherence to SR values when individuals deal with more abstract and prescriptive concepts than when they face a specific issue or case. Therefore, we hypothesize:

#### **H1b**

Attitudes toward *relative*-*SR* are more likely to change under the effects of group activities as opposed to *absolute*-*SR* attitudes.

### Situational and Dispositional Approaches to the Attitude Toward Social Responsibility

Situational and dispositional approaches (Fig. 1) have been well utilized to investigate various human attitudes and behavior. The *situational* approach refers to the importance of the environmental situation in determining attitudes and behaviors (Goldfried and d’Zurilla 1969), while the *dispositional* approach refers to personal characteristics and assumptions in explaining individual attitudes and behaviors (Staw and Cohen-Charash 2005). Because these approaches are often considered to oppose one another, few scholars have chosen both approaches in the same study. In contrast, we employ both the *situational approach* (via group-related factors) and the *dispositional approach* (via personal characteristics) to provide a more robust understanding of individual attitudes toward SR. That is, in our theoretical framework (Fig. 1), we consider two elements that the literature suggests affect individual SR—namely *business ethics attitudes* (which refers to personal beliefs), and *personal characteristics* (intellectual openness). These individual attitudes are then considered as a starting point to understand how change in SR attitudes happen when people are exposed to *group*-*related elements* (such as group sizes, relationships, group “think,” and engagement). These factors are reviewed in the following.

### Attitudes Toward Business Ethics

According to Garriga and Melé (2004), there are SR constructs that specifically examine how ethical beliefs affect behavior. They tend to provide a normative (prescriptive) background for ethics and SR. For this reason, they are named *ethical* approaches to SR. Mainstream approaches in this field usually refer to macro-phenomena such as normative stakeholder theory (Donaldson and Preston 1995), or theories of human rights integration to the business discourse (Barkemeyer 2009). It is worth noting that theories at the macro-level sometimes inform research at the individual level. Carroll’s (1979) pyramid, for example, structures SR in four distinct levels, namely economic, legal, ethical, and discretional responsibilities. These four levels together define and help us to understand the constituent features of SR. More recently, the pyramid has been used to analyze consumer perceptions of organizational SR (Garcia de los Salmones et al. 2005). The operationalization of Carroll’s (1979) construct at the individual level highlights how SR is intertwined with economic and ethical responsibilities (Maignan 2001). These studies seem to suggest that SR is positively related to the ethical dimension (Kolodinsky et al. 2010).

Singhapakdi et al. (1996) and Etheredge (1999) argue that there is, respectively, a strict (either) positive or negative correlation between ethical idealism—i.e., an inquiry into the nature and function of ideals (Rescher 1987)—relativism—i.e., an acceptance of more than a set of basic normal standard (Lyons 1976)—and participants’ perceptions of SR and ethics. Further studies (Kolodinsky et al. 2010; Park 2005) report significant relations between ethics and SR behavior, implying that the former is a pre-condition of the latter. What appears to be particularly striking is that there is a link between individual morality and perceptions of a company’s social performance. Also, some socially responsible companies attract job applicants that are already positively pre-disposed toward SR (Turban and Greening 1997). Given this link, we propose the following hypothesis:

#### **H2**

Individual attitudes toward business ethics affect one’s attitudes toward SR.

### Intellectual Openness

Personal characteristics are defined as stable individual differences that may play a role in relation to one’s attitudes toward SR. They may range from gender (Cheah et al. 2011; Greening and Turban 2000) and age (Nakamura and Watanabe-Muraoka 2006) to personality (Lin et al. 2010; Mudrack 2007).

From a different angle, SR has been related to traditional values (Berkowitz and Lutterman 1968) or conservatism (Mudrack 2007) although results are contradictory. We argue that SR is related to more open traits of individual cognition, values, and personality. This is particularly relevant when seeking to understand SR in the context of child development (Harris et al. 1954). It can be argued that the early development of SR remains with us until we are adults (as a chance-seeking activity; see Bardone 2011). The tendency to be open to change and experience as opposed to being ‘conservative’ can be also framed as intellectual openness (Judge et al. 1999). Drawing on these studies, we claim that the acceptance of SR requires a certain degree of openness for the simple reason that it relates to the external and ever-changing ‘social’ or environmental dimensions of organizational operations. Otherwise stated, some researches provide evidence that there are traits of personality such as ‘openness to experience’ that relate positively to socially oriented attitudes and behaviors (Mudrack 2007; Stasio and Capron 1998). Thus, we propose a relationship between intellectual openness and SR attitudes via the following hypothesis:

#### **H3**

Intellectual openness positively affects individual SR attitudes.

### Group-Related Factors

Complex and uncertain environments force people to co-operate more, thus groups and teams flourish in today’s working environments (Li and Cropanzano 2009). Each group establishes specific dynamics that may vary depending on size, individual skills and capabilities, gender composition, social relations between group members, and more (Bettenhausen 1991; Kerr and Tindale 2004). In order to further develop theory in this area, this study establishes a rationale to understand how group dynamics affect SR.

First, group size is an important variable when seeking to understand dynamics and it has been long studied. Groups with larger numbers of people tend to be less efficient in the use of time and resources, but achieve better results (Cohen and Bailey 1997). However, size may only be a moderating factor in the context of this study since larger groups usually hold more stringent expectations in terms of social norms and values (Shepperd and Wright 1989). In addition to that, larger groups account for greater deviance among members (Moreland et al. 1996); this means that interactions among members are less frequent (Steiner 1972), conflict becomes more likely (Valacich et al. 1995), and communication quality decreases independent of the means used (Chidambaram and Tung 2005; Lowry et al. 2006). This points to the fact that it may take more time for individual members to be influenced by wider group dynamics. Therefore,

#### **H4**

The larger the group size, the less likely it is for individuals to change their attitudes toward SR.

Second, group members may be affected by previous or existing social relationships among each other (Moreland and Levine 2002). Members who know one another and believe there is some level of ‘closeness’ (e.g., friendship or other relation) may find it easier to either impose their opinion on the group (if it is similar) or to adapt (if dissimilar; Karau and Williams 1993). Knowing each other may also lead group members to develop a ‘feel-good’ attitude toward the group which makes group work run more smoothly (Levine and Moreland 1990). Given that previous or established knowledge of group members helps people work well together and develop a positive attitude, we argue that this mechanism could influence how members adapt their attitudes toward SR; hence.

#### **H5**

The *strength of social relationship* among group members positively affects the change of SR attitudes.

Finally, engagement (Saks 2006) has been identified as an important element for the analysis of group behavior (Miles and Kivlighan 2012). Engagement can be interpreted as the group member’s commitment to actively influence shared knowledge (Miles and Kivlighan 2012; Saks 2006). Group engagement can be related to climate (Colquitt et al. 2002), which is defined as “psychologically meaningful moral descriptions that people can agree characterizes a system’s [set of] practices and procedures” (Schneider 1975, p. 474). When work climate is inclusive (Nishii 2013) and it is formulated by group members such that everyone is perceived as making a fair contribution, then performance and outcomes improve (Colquitt et al. 2002). Thus, so-called climate is likely to lead to increased engagement (Miles and Kivlighan 2012) and convergence of how opinions are shared within the group (Moreland and Levine 2002). Thus, we hypothesize:

#### **H6**

Higher group engagement increases the likelihood that individual attitudes toward SR change as an effect of group activities.

Drawing on this perspective, group activities may serve as a moderator, with a caveat. We argue that groups develop something called ‘tendency toward the mean.’ This tendency affects individual beliefs with an impact on members that is similar and depends on (is a function of) how the idea is shared by group members (Levine and Moreland 1990; 2004). In other words, the interactions among group members are vital for any type of group operations (Senge 2006). Hence, group members change or stick to their opinions depending on how group interactions relate to their initial opinion. This leads to the following:

#### **H7a**

Individuals that highly value SR are more likely to stick to their initial opinion.

#### **H7b**

Higher levels of group SR positively affect individual change of attitudes toward SR.

## Method

### Sample

The data were collected from final year business students in the UK. A total of 469 students representing 26 nationalities participated in the study. Data were collected in two waves: 276 participants in wave one, year one, and the remaining participants in wave two, year two. The study in year two was an exact replica of that undertaken in year one. Participants were allocated to 105 groups; the average group size in the experiment was 4.59 (*sd* = .81). A measure of relationships among participants shows that most of them either have met a few times before, or only met at the university, i.e., participants were mostly unacquainted (*mean* = 2.64, *sd* = 1.12; the value ‘1’ *never met before* to ‘7’ *close friends*). The choice of student participants for this particular study is partially justified by the exploratory nature of the study; most of them had held one or more jobs for over one year, had a one-year internship within their business program, and were likely to have experienced group work before (either in business or academic contexts). A very large proportion, 97 % of participants, had work experience (*mean* = 3.25 years) in one or more industries (*mean* = 1.69, *sd* = .95). Slightly less than half, 47 % of participants were female. The mean age of all participants was 22.4 (*sd* = 2.58) and the majority, 87 % of respondents, defined themselves as British. To capture the sample’s potential differences in ethical values, we asked for religious affiliation and found that 49 % defined themselves as Christian or Catholic, around 2 % as Muslims, and the remaining declared to have no religious affiliation whatsoever. A significant number of participants, 75 % (*sd* = .43), declared they had a basic understanding of SR, gained from previous university courses.

### Design and Procedure

Participants were briefed on a case. This focused on a large multinational pharmaceutical company that made the decision to recall their cleaning solution for contact lenses (Appendix 1). The decision came after a limited number of customers in a few countries—including Singapore, Hong Kong, and the USA—reported loss of vision or suffered serious eye injuries. Participants were not given the final decision, and were asked to offer some alternatives regarding what the company could have done to address the issue.

The procedure was designed to take place in two phases. First, participants were subjected to a survey after the case briefing. The first part of the survey contained a section for personal and demographic data, general SR attitudes, business ethics, and SR related to the case. This part was completed by individual participants. In the second phase, participants were allocated into small groups to discuss the case again. The average time it took the group to complete the assignment was 22.19 min (*sd* = 9.04). After the discussion, participants were asked to answer further questions on group demographics, group activity, and, again, on SR. The time lag between the two phases was one week. It was reasoned that this interval was appropriate in that it was not too short to recall exactly what participants did, and not too long for them to forget about the case.

### Measures

All measures were tested for internal reliability with Cronbach’s α (Nunnally 1978) and McDonald’s *ω*_*h*_ (McDonald 1999). The difference between the way the two indicators are developed suggests that information on the “proportion of scale variance due to a general factor” (Zinbarg et al. 2005, p. 132) is missing when only the traditional α is used. McDonald’s *ω*_*h*_ is also described as a (hierarchical) measure of one-factor saturation of a test (Zinbarg et al. 2005). Since the items of many of the scales load on a single general factor, we provide both measures when that is the case. All items for scales used in this study are reported in Appendix 2.

#### Attitudes Toward Business Ethics

We used the scale provided by Reidenbach and Robin (1990) to measure general attitudes toward business ethics, making sure that participants referred to the business case when scoring on the items. The scale consists of eight items, assessed on a Likert scale ranging from ‘1’ to ‘7’ that asks participants to assess the company’s behavior on several issues such as ‘fairness’, ‘moral acceptance,’ and ‘cultural acceptability.’ The scale was measured twice, before and after group discussion. Cronbach’s α for the construct is .92 before and .93 after the group exercise. McDonald’s *ω*_*h*_ is .83 for this variable.

#### Attitudes Toward SR

This variable is measured using two scales, a *relative*-*SR* attitude that deals with *ad hoc* situations, problems, and issues, distinct from a more abstract and general *absolute*-*SR* that tends to refer to what one believes all businesses should/must do. A measure of the former is provided by adapting the work of Garcia de los Salmones et al. (2005) to the current study. Following these author’s footsteps, we used this scale to understand how participants perceived SR relative to a company’s behavior. Participants were asked to assess their perception of economic, legal, and ethical responsibilities via three, two, and four items, respectively (Appendix 2). The measure reported a Cronbach’s α of .82 and McDonald’s *ω*_*h*_ of .78 before group activities and .79 and .70, respectively, after.

Together with the mini-case, we wanted to test general or *absolute* attitude toward businesses’ SR. The decision to develop a new measure was due to the fact that (a) most SR measures are embedded with ethics measurements, and (b) they are associated with a particular theory or model of CSR. In order to do so, we developed a new multi-item scale using a characteristics checklist, a method often used in applied psychology to measure attitudes or traits (e.g., Ajzen 2005; Watson and Clark 1994). After a review of other measures of SR, we developed a number of items for our list that, following a round of consultation with experts, was reduced to 12 (see Appendix 2 for the full list and to have more details on how the scale was developed). Participants were asked to assess each of the items as core values of any operating businesses using numbers from ‘1’ to ‘7’, with ‘1’ being *non*-*existent/irrelevant* and ‘7’ being *core value/totally relevant*. *Absolute*-*SR* is framed as the understanding of the values and principles defining the role (function) of businesses in society. The role or function of business in society has long been used to provide arguments to legitimize or delegitimize CSR, mostly by ‘utilitarian’ (Secchi 2007) or ‘instrumental’ (Garriga and Melé 2004) theorists. A famous stance is taken in an article by Milton Friedman in the New York Times Magazine (1970), in which he argued that the only responsibility of business is to maximize its profits. The line of arguments was out of economic and social functionalism. In addition, he answered the question “what is the role of operating businesses” very straightforwardly. For him, the core values of businesses could only be economic or financial, because that is what is needed to fulfill the firm’s social role.

That debate is long gone now, although others articulate their reasoning along similar but milder lines (e.g., Porter and Kramer 2002). However, as the literature shows, the debate is no longer as polarized as it was, and scholars seem to accept that there are multiple roles and values that companies stand for (e.g., Freeman et al. 2004). To ask participants about the core values of businesses should allow us to understand what the underlying assumptions are that people make about whether businesses should look at some prevalent economic motives or be proactive social agents. In summary, an assessment of the “core values” of businesses should provide a good indication of what participants think the role/function of business is.

In addition, in a standard scale construction, it is extremely important that the objective of the measurement is not too apparent. This is necessary in order to guarantee that subjects are not primed or put in a disposition that is particularly keen on favoring a social desirability (or any other type of) bias (Podsakoff et al. 2012). This is why we considered that referring to “core values” of businesses is a good proxy for understanding what one thinks the role of business in society is. Since we intend to measure social responsibility, the statements we selected for this measure represent items that strictly relate to that literature.

A CFA shows some support for a one-factor model (*χ* ^2^ = 113.87[*df* = 43], *p* < .001; CFI = .973; RMSEA = .059; SRMR = .034; BIC = 329.14). We also fitted a three-factor model to the construct (*χ* ^2^ = 123.58[*df* = 38], *p* < .001; CFI = .963; RMSEA = .069; SRMR = .038; BIC = 295.79), grouping items on ‘business responsibilities’ (items 1, 2, 4, and 11), ‘community orientation’ (items 5, 6, 7, and 9), and ‘good citizenship’ (this can also be ‘legal responsibilities’; items 3, 8, and 12). Comparing the indices of good fit for the two models, we conclude that the one-factor model is slightly better, although the three-factor model also fits the data well. A Cronbach’s α of .90 and .88 and McDonald’s *ω*_*h*_ of .74 and .68, respectively, before and after group ‘treatment’ were recorded.

We also conducted validity tests to check whether this measure is different from other measures of SR; namely Reidenbach and Robin’s (1990) business ethics scale and Garcia de los Salmones et al. (2005)—what we labeled *relative*-*SR* scale. We found that Pearson’s correlation coefficient with *relative*-*SR* and *business ethics attitudes* is respectively .228 (*p* < .001) and .029 (*p* = .52). Subsequent to exposure to group work, these measures demonstrate similar but weaker patterns of correlation: .119 (*p* < .05) for *absolute*- and *relative*-*SR*, and −.018 (*p* = .70) for business ethics and *absolute*-*SR*. Following Farrell (2010), the shared variance (i.e., the amount of variance that one variable explains of another variable) is given by the squared correlation. In our cases, we have .049 prior to and .014 subsequent to group work. After checking for the attenuation-corrected coefficients (Cohen et al. 2003, pp. 56–57), we found enough evidence of *discriminant validity* for the construct (DeVellis 2012).

#### Intellectual Openness

During the first phase, we tested participants for intellectual openness to study whether this attitude relates to either individual SR or its change after group activity. A ten-item scale of intellectual openness from Jackson, Paunonen, and Trembley (2000) was measured on a Likert scale, with values ranging from ‘1’ *strongly disagree* to ‘7’ *strongly agree*. Cronbach’s α for the construct is .69, and McDonald’s *ω*_*h*_ is .47. Despite the second value is low, this measure was tested several times in the psychology literature and we decided to use it in this study.

#### Group-Related Variables

*Group size* was simply assessed by the number of participants that worked in a given group and we also recorded participants’ self-reported data for the *length of the discussion*. The *strength of social relationships* (or level of acquaintance) among group members was also assessed by asking each participant to rank it on a seven-point scale, with ‘1’ being *never met before*, and ‘7’ being *we have always known each other*. We used a cumulative measure providing the average *strength of social relationships* for each participant in respect to their group. Group climate was measured as the level of *engagement* in the discussion. It was assessed using three items (Appendix 2) on a seven-point scale, with ‘1’ being *strongly disagree* and ‘7’ *strongly agree*. The creation of an *ad hoc* measure for engagement is due to the particular conditions of the experiment that do not match existing measures of employee engagement (Harter et al. 2002). CFA shows that a one-factor construct fits the data well (*χ* ^2^ = 3.65[*df* = 2], *p* = .16; CFI = .996; RMSEA = .042; SRMR = .015). Coefficient α and McDonald’s *ω*_*h*_ for the construct are both .72.

To capture the impact of group activities on individual attitudes toward SR, we used the *weight of group activities* (hereafter, WOGA) calculated for each of the measures of SR (i.e., *rel*, company-related; *abs*, absolute) and business ethics (*be*). The WOGA is the difference between individual pre-group and post-group assessments of SR weighted on the difference between *emergent group thinking* (average SR attitudes of each group member) and the individual pre-group assessment. Both numerator and denominator differences are taken at their absolute value: |*SR*_*ik1*_ − *SR*_*ik0*_|/|*G*-*SR*_*k*_ − *SR*_*ik0*_|, where *SR* is social responsibility, *i* is a participant and *k* indicates the group one is affiliated to at time 0 (pre-group test) and at time 1 (post-group test); and *G*-*SR* is the average value of SR attitudes of all group members, i.e., the post-group SR attitudes. A similar calculation has been used in studies of advice discounting (Gino and Moore 2007; Harvey and Fisher 1997). When WOGA is 0, it means that group activities registered no impact in the change of individual attitudes toward SR and either one’s attitude registered no change at all (the numerator is zero) or the change is not due to the group activities (denominator is zero). If WOGA is >0 it means that there has been some change in attitudes and the measure gives a value of SR change that is linked to the group. Assuming that there is a difference in the value of SR between pre- and post-group activities, this value is discounted on the “distance” that the initial SR value (*SR*_*ik0*_) has on the average group attitudes toward SR (G-*SR*_*k*_). This provides a weighted value for change and tells us how much impact the group has had on one’s attitude change. Assuming that |*SR*_*ik1*_ − *SR*_*ik0*_| < |*G*-*SR*_*k*_ − *SR*_*ik0*_| may mean that a relatively small change in individual attitudes is large compared to the “distance” that the member had to cover to adapt to the group average. Instead, if |*SR*_*ik1*_ − *SR*_*ik0*_| > |*G*-*SR*_*k*_ − *SR*_*ik0*_| then the overall change is not entirely due to group activities; instead the member has changed somehow independently from the group. The peculiarity of this equation is that it does not discern positive (upward) from negative (downward) changes but only provides a weight or multiplier factor for the group impact on individual changes in attitude.

The *change in individual attitudes* is used as a dependent variable to test hypotheses 4–7 and, for this reason, it does not need to be ‘discounted’ by the group effects. This is done to avoid that the effect of the group in the regression appears as a result of how the dependent variable is constructed. For this reason, it is calculated by *post*-*group SR* (AGSR)—*pre*-*group SR* (BGSR) divided by the attitude recorded before the group activity (BGSR). We feared that the square of the differences may lead to too much weight on change, thus biasing the results. The formula for attitude change measurement (*ACM*) is *ACM*_*relSR*_ = (*AGSR*_*rel*_ − *BGSR*_*rel*_)/*BGSR*_*rel*_ and *ACM*_*absSR*_ = (*AGSR*_*abs*_ − *BGSR*_*abs*_)/*BGSR*_*abs*_.

#### Control and Other Variables

Most of the coding for the demographic variables was standard: *Gender* has been coded as ‘1’ for female and ‘0’ for male; *groups* are numbered progressively, from 1 to 105; *age* is measured in years; *nationality* is measured as dummy with ‘1’ being British and ‘0’ everyone else, due to a smaller number of overseas participants; *tenure* or seniority is measured in years; and *work experience* is given by the number of different sectors within which participants worked. Further questions assessed *religion* (categorical) and previous *exposure to SR* (also labeled *existing CSR knowledge*), with ‘1’ indicating that they were aware and ‘0’ indicating no awareness of SR. Some of these variables were used as control, others to conduct checks on the data and have a clearer idea of the shape of the sample.

### Analytical Strategy

Eight participants were deleted from the original sample as a result of more than 20 % of missing data in their respective rows; the final sample size is *N* = 461. Following Roth (1994) and Cohen et al. (2003), we handled missing data with the aim of preserving standard deviation for each given measure. Therefore, we used mean substitution for missing data up to 2 % and OLS regression for those exceeding 2 % of values in the column (item).

The analytical strategy for the study uses OLS regressions to test hypotheses 2 and 3, where the analysis is not affected by the group. When individual and group effects are nested together so that the independence of observations is violated, the analysis is conducted using a multilevel random coefficient model (MRCM; Hofmann 1997; Raudenbush and Bryk 2002). This technique enables the structuring of the analysis at the individual and group level, and provides better parameter estimation (Bliese and Hanges 2004). MRCM, also referred to as ‘hierarchical linear modeling’ (Raudenbush and Bryk 2002), has been used to deal with problems arising with clustered data (Cohen et al. 2003; Kenny et al. 2002). In order to justify the use of MRCM, the variance of the dependent variables has to depend on group-related effects (Bliese 1998; 2000). Thus, using the inter-correlation coefficient (ICC) we find that the group variance is .31 for relative-SR variation (ACM_relSR_) and .14 for absolute-SR variation (ACM_absSR_). The cumulative measure (ACM) shows an ICC that is .26. In addition to the estimation of variance, we need to define whether this is significantly different from zero. This value is the variance of the intercept term in a model (the null model) that estimates each of the dependent variables based solely on the intercept term with groups used in the random component of the regression equation. In order to understand whether the variance of the intercept is significantly different from zero, we compare this null model with another that does not include groups in its random component and estimation is done via a generalized linear model (i.e., without a random intercept). We compare these two models per each dependent variable using an ANOVA procedure. With the variable ACM_relSR_, the random intercept model appears significantly different from zero (Δ – 2 log-likelihood = 127.444; *p* < .001). For the dependent variable ACM_absSR_, the random intercept model is also significantly different from zero (Δ – 2 log-likelihood = 54.915; *p* < .001). The cumulative variable ACM_SR_ confirms the trend, showing that the random model is preferred (Δ – 2 log-likelihood = 39.679; *p* < .001). The three tests demonstrate that a random intercept provides a better estimate than a model without the random component. The equation to estimate the model is of the type

$$Y_{ij} = \beta_{0j} + \beta_{ij} X_{i} + r_{ij}$$

with *β*_0*j*_ being the intercept that is a function of a fixed intercept and of group-level coefficients, *β*_*ij*_ is a function of either fixed or random coefficients that depend on the estimate, *X*_*i*_ is the set of variables at the individual level, and *r*_*ij*_ is the error term. After substituting the terms with the variables of this study, the combined two-level equation is

$$\begin{aligned} {\text{ACM}}_{ij} = \gamma_{00} + \gamma_{0 1}\left( {{\text{group relative-SR}}} \right) + \gamma_{0 2}\left( {{\text{group absolute-SR}}} \right) + \gamma_{10}\left( {{\text{relative-SR}}} \right) + \hfill \\ \gamma_{1 1}\left( {{\text{relative-SR}}} \right) \, * \, \left( {{\text{group relative-SR}}} \right) + \gamma_{20}\left( {{\text{absolute-SR}}} \right) + \gamma_{2 1}\left( {{\text{absolute-SR}}} \right) \, * \, ({\text{group}} \hfill \\ {\text{absolute-SR}}) + \gamma_{30}\left( {\text{intellectual openness}} \right) + \gamma_{40}\left( {\text{group size}} \right) + \gamma_{50}\left( {\text{acquaintance}} \right) + \hfill \\ \gamma_{60}\left( {\text{group engagement}} \right) + u_{0j } + u_{1j } + u_{2j } + r_{ij} \hfill \\ \end{aligned}$$

In this MRCM, *u*_0*j*_, *u*_1*j*_, and *u*_2*j*_ represent the error terms of the random coefficient equations (fixed coefficient equations do not have error terms). Some warnings about endogeneity issues with multilevel modeling have been raised (Leeuw and Meijer 2008) so, we thoroughly tested the mathematical formulation of the model and its results with Hausman’s test (1978) and found this problem to be not significant. The same equation is used for ACM_absSR_, ACM_relSR_, and also to estimate parameters for the combined effects of group and individual levels on absolute and relative-SR taken together (cumulative SR or ACM_SR_). In the procedure, we regress ISR change on initial ISR values, among other variables, consistently with the existing literature on the analysis of change, whether it is individual (e.g., Gino and Moore 2007) or socio-economic (e.g., Barro 1991). Statistical analyses are conducted using *R* version 3.1.3 (R Core Team 2015), an open source software for statistical analysis.

## Results

### Descriptive Statistics

Table 1 presents descriptive statistics (means, standard deviations, and correlations) for the variables used in the study. As already anticipated in the previous section, there are a few variables showing significant and strong correlations, except for group size and assessed activity (*r* = .83). Since the two measurements are not used together in the analysis because a combination of these is used instead, this is not an issue in the analysis. As a check for all regression analyses of this study, we performed variance inflation factor (VIF) tests for multicollinearity. Results showed values around 1 for each variable, well below the threshold (Cohen et al. 2003).

**Table 1:** Descriptive statistics: Means, standard deviations, and Pearson’s correlation coefficients

Variable	1	2	3	4	5	6	7	8	9
1 Age									
2 Gender	0.01								
3 Group size	−0.13**	−0.09*							
4 Existing CSR knowledge	−0.11*	0.02	0.11*						
5 Intellectual openness	0.07	−0.04	−0.04	0.25***					
6 Bus. ethics attitudes	−0.08^†^	−0.15**	0.06	0.02	−0.03				
7 Relative-SR	−0.04	−0.02	0.06	−0.01	0.03	0.54***			
8 Absolute-SR	−0.04	0.17***	−0.04	−0.04	0.11*	−0.01	0.17***		
9 Bus. ethics attitudes AG	−0.07	−0.08^†^	0.11*	−0.01	0.04	0.36***	0.22***	−0.02	
10 Relative-SR AG	−0.08^†^	−0.04	0.08^†^	−0.04	0.07	0.34***	0.47***	0.12*	0.49***
11 Absolute-SR AG	−0.03	0.21***	−0.15***	−0.06	0.09*	−0.09*	−0.01	0.52***	−0.02
12 Strength of relations	−0.13**	−0.03	0.07	0.08	0.05	0.02	−0.05	0.13	−0.03
13 Length of discussion	0.09^†^	−0.03	−0.07	−0.00	0.11*	0.07	0.08^†^	−0.01	−0.01
14 Group engagement	−0.03	0.08	−0.06	−0.02	0.18***	−0.08^†^	0.01	0.18***	0.03
15 Assessed activity	−0.05	−0.05	0.83***	0.12*	0.04	0.06	0.01	−0.07	0.07
16 ACM—relative-SR	−0.06	0.01	−0.01	−0.03	0.04	−0.17***	−0.44***	−0.03	0.25***
17 ACM—absolute-SR	−0.00	−0.01	−0.10*	0.01	−0.04	−0.12*	−0.22***	−0.60***	−0.01
Mean	22.39	0.47	4.61	0.75	4.61	3.24	4.28	5.42	3.69
Standard deviation	2.58	0.50	0.78	0.43	0.58	1.10	0.83	0.86	1.18

Variable	10	11	12	13	14	15	16	17
1 Age								
2 Gender								
3 Group size								
4 Existing CSR knowledge								
5 Intellectual openness								
6 Bus. ethics attitudes								
7 Relative-SR								
8 Absolute-SR								
9 Bus. ethics attitudes AG								
10 Relative-SR AG								
11 Absolute-SR AG	0.11*							
12 Strength of relations	0.12**	−0.00						
13 Length of discussion	0.04	−0.00	0.09^†^					
14 Group engagement	0.02	0.22***	0.14**	0.14**				
15 Assessed activity	0.05	−0.15**	0.14**	−0.07	−0.09^†^			
16 ACM—relative-SR	0.55***	0.14**	0.07	−0.02	0.01	0.00		
17 ACM—absolute-SR	−0.06	0.31***	−0.01	−0.01	0.01	−0.07	0.16***	
Mean	4.37	5.53	2.64	22.29	6.03	4.61	0.04	0.04
Standard deviation	0.92	0.78	1.12	8.98	0.74	0.76	0.23	0.21

*Note: * Strength of relations is calculated as the individual average of the acquaintance level with each member of the group; *AG * after group activities

Significance codes: *** *p* < .001, ** *p* < .01, * *p* < .05, ^†^ *p* < .1

### Tests of Hypotheses

H1a postulates that there is a positive correlation between *relative*-*SR* and *absolute*-*SR* and that the two constructs are different. This issue has been discussed above, when assessing discriminant validity for *absolute*-*SR*. Table 1 shows that the two measures are correlated both in the pre- (.17) and in the post-group activities (.11). A *t* *test* for the difference of means shows that the null hypothesis is rejected (*t* = –20.44[*df* = 919], *p* < .001; these are results obtained for measures in the pre-group activities; post-group results are very similar and not reported here). The two measures are different; H1a is therefore supported.

Under H1b, we compare *relative*-*SR* and *absolute*-*SR* variability after individuals are exposed to group activities. We hypothesize that relative (case-related) attitudes are more subject to change than the attitudes governed by abstract and general constructs. This is tested using the two measures WOGA_*rel*_ and WOGA_*abs*_ that are instrument tools which assess the weight of group activities over the change of opinions. Another *t* *test* provides us with enough information (*t* = −1[*df* = 460], *p* = .32) necessary to conclude that H1b is rejected. There is no significant difference concerning how group activity influences individual attitudes toward both *relative*-*SR* and *absolute*-*SR*. As shown below, this result does not mean that there is no group effect on ISR change or that the result is irrelevant. All that the rejection of H1b tells us is that the ways in which *absolute*-*SR* and *relative*-*SR* change does not seem to differ.

H2 assumes that there is a positive relation between ethical judgement on business, and both constructs of *relative*-*SR* and *absolute*-*SR*. Repeated measures—i.e., pre- and post-group activities—are highly correlated, as expected (Table 1). We know from the section above that these two SR constructs are different, although *business ethics* more strongly correlates with *relative*-*SR* (.54) than with *absolute*-*SR* (–.01), suggesting we probably found a way to distinguish SR from ethics more neatly at the individual level of analysis. Table 2 presents results of an OLS regression that tests the extent to which business ethics affects SR. We tested the hypothesis using a combination of *absolute*-*SR* and *relative*-*SR* as dependent variables, and then observing the impact on each SR individually. Model 1, Model 3, and Model 5 (Table 2) present control variables, including the personality trait *openness to experience*. From Model 2 we observe that the variable *business ethics* predicts higher SR (*β* = .44) with significantly more variance explained (∆R^2^ = .13) than the model with control variables only (Model 1). When we split SR into the two components, we find that only *relative*-*SR* is positively affected by *business ethics* (*β* = .42), and it is also the only significant variable in Models 3 and 4. Models 5 and 6 indicate that *absolute*-*SR* is not affected by *business ethics* (*β* = .01, *p* = .70), with *intellectual openness* (*β* = .22) and *gender* (*β* = .32) showing positive beta coefficients. H2 is thus supported, with a strong effect exercised by *business ethics* on *relative*-*SR*.

**Table 2:** OLS regression results for Business Ethics, Intellectual Openness, and Existing CSR Knowledge on Social Responsibility

	Model 1 DV: abs+rel SR	Model 2 DV: abs+rel SR	Model 3 DV: rel SR	Model 4 DV: rel SR	Model 5 DV: abs-SR	Model 6 DV: abs-SR
(Intercept)	9.272*** (0.684)	7.345*** (0.674)	4.432*** (0.446)	2.568*** (0.395)	4.840*** (0.447)	4.777*** (0.475)
Age	−0.036 (0.023)	−0.021 (0.022)	−0.016 (0.015)	−0.002 (0.013)	−0.020 (0.015)	−0.019 (0.015)
Gender	0.276* (0.119)	0.424*** (0.112)	−0.036 (0.078)	0.107 (0.066)	0.312*** (0.078)	0.317*** (0.079)
Existing CSR knowledge	−0.240^†^ (0.145)	−0.262^†^ (0.134)	−0.056 (0.094)	−0.078 (0.079)	−0.183^†^ (0.095)	−0.184^†^ (0.095)
Intellectual openness	0.278** (0.106)	0.304** (0.099)	0.059 (0.069)	0.084 (0.058)	0.219** (0.069)	0.220** (0.070)
Business ethics attitudes		0.438*** (0.051)		0.424*** (0.030)		0.014 (0.036)
*R* ^2^	0.030	0.164	0.004	0.308	0.055	0.055
∆ * R* ^2^	–	0.134	−	0.304	−	0.000
*F*-test	3.478	17.883	0.508	40.444	6.647	5.340
*p* value	0.008	0.000	0.730	0.000	0.000	0.000
*N*	461	461	461	461	461	461

Significance codes: *** *p* < .001; ** *p* < .01; * *p* < .05; ^†^ *p* < .1

The remaining hypotheses are tested with MRCM regressions presented in Table 3. Models 1 to 3 in Table 3 indicate no strong relationships between intellectual openness and SR attitude change (γ_30_ = –.000, Model 3), group size, and SR attitude change (γ_40_ = –.016, Model 3), or strength of social relationship among group members (γ_50_ = .005, Model 3). Thus, H3, H4, and H5 are rejected.

**Table 3:** Results of the Multilevel Random Coefficient Model (MRCM) Regressions

	Model 1	Model 2	Model 3
	DV: ACM_*rel*_	DV: ACM_*abs*_	DV: ACM_*SR*_
(Intercept)$\gamma _{00}$	0.043 (0.070)	−0.071 (0.062)	−0.027 (0.096)
Relative-SR $\gamma _{10}$	−0.175*** (0.009)	−0.023** (0.008)	−0.198*** (0.012)
Absolute-SR $\gamma _{20}$	0.009 (0.008)	−0.179*** (0.008)	−0.170*** 0.012
Group relative-SR $\gamma _{01}$	0.222*** (0.010)	0.005 (0.009)	0.228*** (0.014)
Group absolute-SR $\gamma _{02}$	0.010 (0.014)	0.179*** (0.012)	0.190*** (0.019)
Intellectual openness $\gamma _{30}$	−0.008 (0.011)	−0.008 (0.010)	−0.000 (0.015)
Group size $\gamma _{40}$	−0.008 (0.009)	−0.007 (0.008)	−0.016 (0.012)
Strength of relations $\gamma _{50}$	0.003 (0.006)	0.002 (0.005)	0.005 (0.008)
Group engagement $\gamma _{60}$	0.007 (0.009)	0.022** (0.008)	0.030* (0.013)
Relative-SR × Group rel SR $\gamma _{11}$	−0.036*** (0.010)	0.013 (0.009)	−0.023^†^ (0.014)
Absolute-SR × Group abs-SR $\gamma _{21}$	−0.016 (0.014)	−0.069*** (0.013)	−0.085*** (0.020)

Significance codes: *** *p* < .001; ** *p* < .01; * *p* < .05; ^†^ *p* < .1

*Group engagement* is found to be positively associated with *absolute*-*SR* (γ_60_ = .022, Model 2), and the cumulative measure of SR change after group activities (γ_60_ = .030, Model 3) although it is unrelated to *relative*-*SR*. The effect on a combined SR change is still positive and significant (*p* < .05 level) even if one of the two legs of SR change (*relative*-*SR*) has a non-significant coefficient. Hence, the overall effect is still strong despite being completely related to the impact of *absolute*-*SR* on cumulative ISR change. It is indisputable that an impact of some ISR change is in place and H6 is therefore accepted. H7a states that individuals with higher appreciation of SR are more likely to stick to their initial opinions. Table 3 shows that both *absolute*- and *relative*-*SR* are negatively related to *attitude change measurement* (ACM; γ_10_ = –.198; γ_20_ = –.170 in Model 3). In other words, the higher the initial set of attitudes toward SR, the more one is likely to change his or her opinion. The negative coefficient indicates a negative slope in the regression line; hence, the higher initial attitudes toward SR, the lower the change. H7a is thus accepted.

H7b suggests that higher levels of group social responsibility positively affect individuals’ change of attitudes toward SR. In Model 3 there is a positive and significant impact of the group on ACMSR (γ_01_ = .228; γ_02_ = .190). The same effects are found in the other two models, with *group relative*-*SR* (γ_01_ = .222) and *group absolute*-*SR* (γ_02_ = .179) that appear to be significant in Model 1 and Model 2, respectively. There is a positive effect of groups on the individual change of attitudes that suggests H7b is accepted. A further consideration of these relationships (Table 3) indicates that there is an impact of how the group affects ISR as it predicts the change of attitudes (ACM_*rel*_, ACM_*abs*_, and ACM_*SR*_). The first finding is that there is a moderation effect, although very small, and group SR variations affects the relationship between the two variables in all three models (γ_11_ = –.036, Model 1; γ_11_ = –.023, *p* < .1, Model 3; and γ_21_ = –.069, Model 2; γ_21_ = –.085, Model 3). However, the direction of the coefficients is always negative, meaning that group SR affects those individuals with lower levels of SR and, therefore, provides additional ground for accepting H7b.

**Fig. 2:**
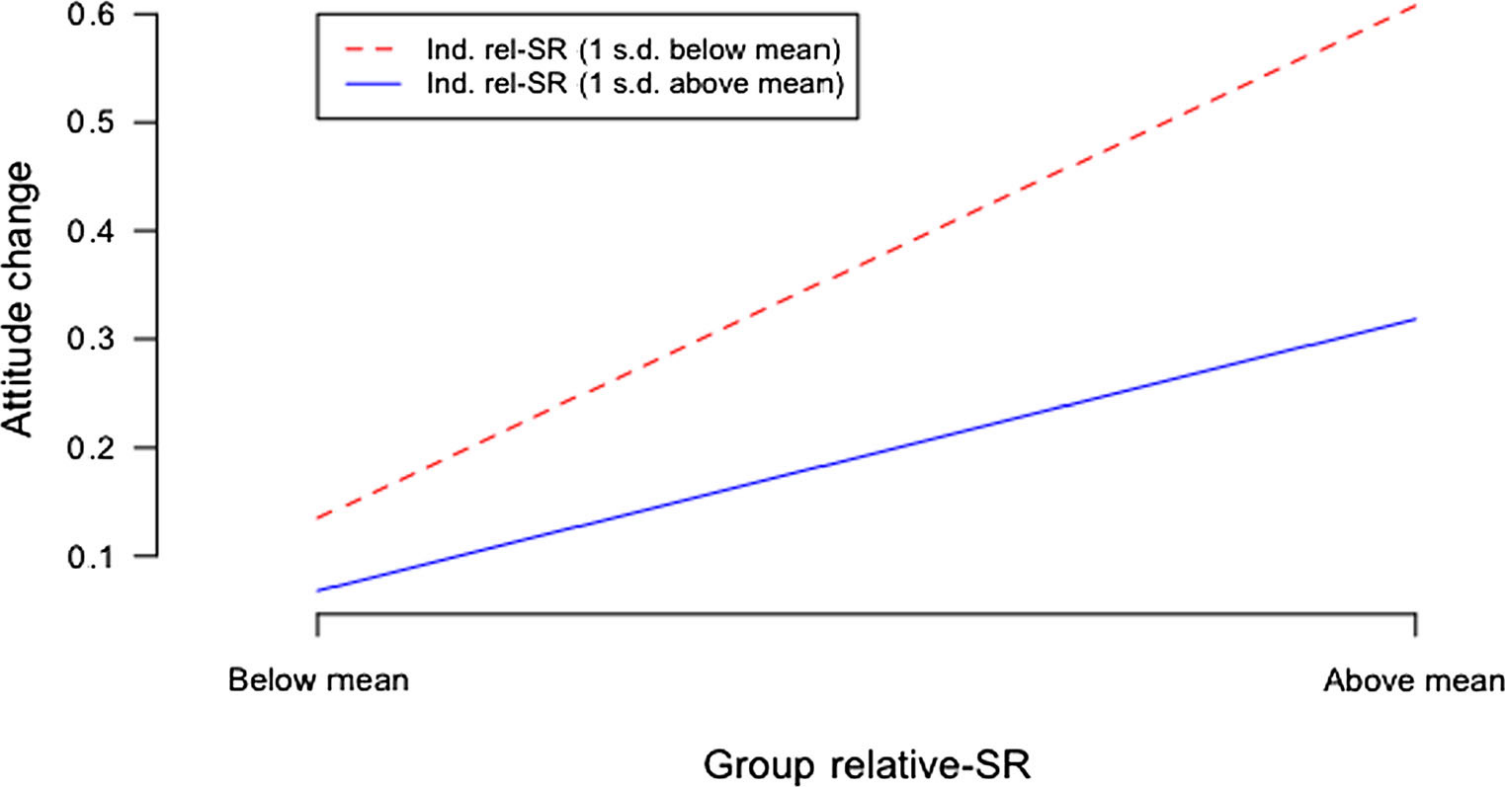
Interaction plot of the effect of group relative-SR on the relationship between the individual relative-SR and attitude change mechanism (ACM)

**Fig. 3:**
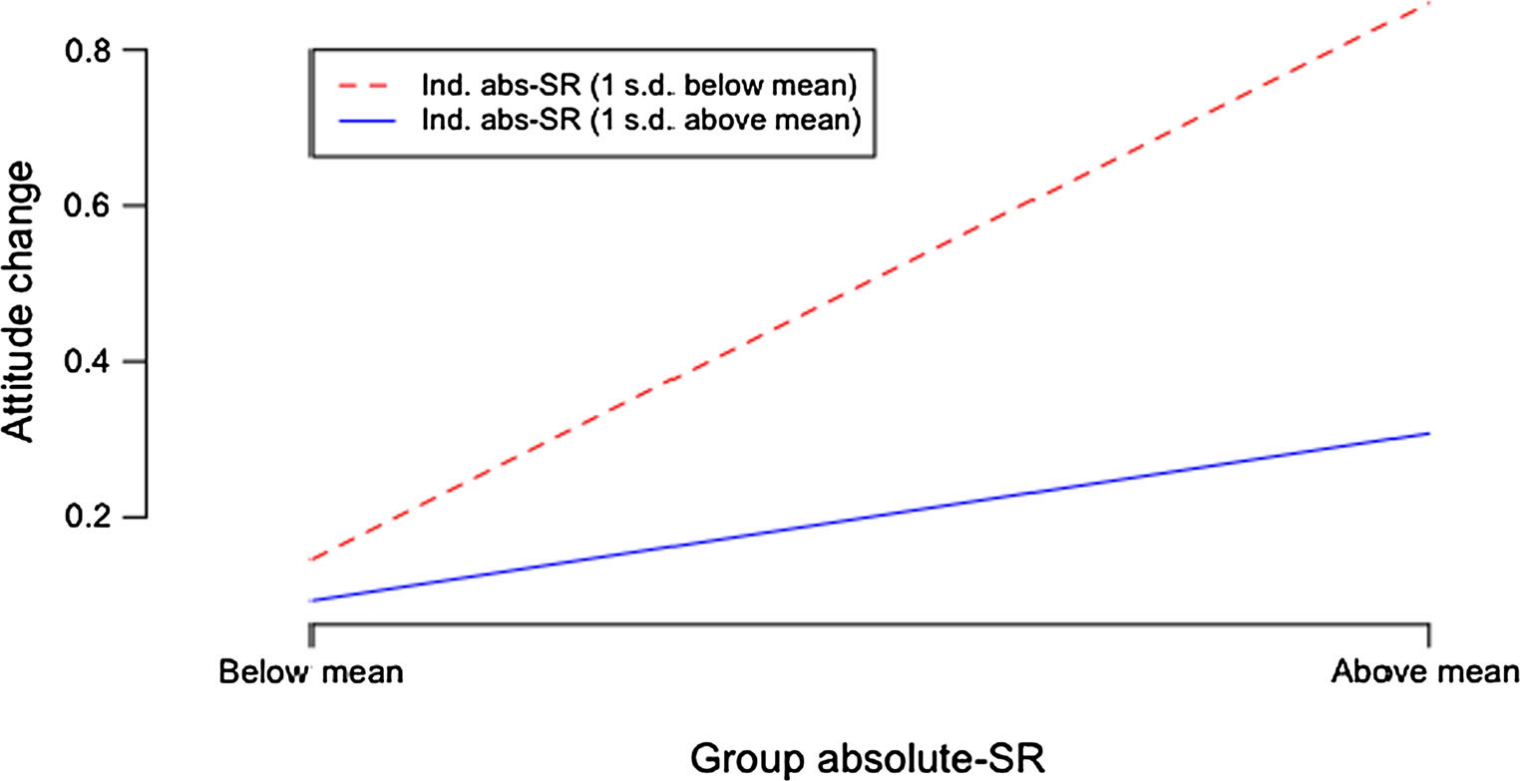
Interaction Plot of the effect of group absolute-SR on the relationship between the individual absolute-SR and attitude change mechanism (ACM)

To further support these results, Figs. 2 and 3 present interaction plots of two classes of individuals: (a) those with low initial social responsibility (dotted red line), and (b) those with high initial social responsibility (solid blue line). The two figures show individual *relative*-*SR* and *absolute*-*SR*, respectively. The two classes are plotted against low and high social responsibility of their respective groups, and attitude toward change (ACM). What emerges from the two plots is that individuals with below-mean SR show the highest change when the group-level SR goes from low to high, while people who already have above-mean SR do not change much. This strengthens our findings for H7a and H7b.

Table 4 summarizes all the hypotheses and their results. We explain our findings in the discussion part below.

**Table 4:** Summarizing the outcomes of the hypotheses

Hypotheses	Results
**H1a**: There is a more practical attitude toward SR, or *relative-SR*, which is correlated with but different from a more abstract SR attitude, or *absolute-SR*	Supported
**H1b**: Attitudes toward *relative-SR* are more likely to change under the effects of group activities as opposed to *absolute-SR* attitudes	Rejected (The way the two change does not seem to differ)
**H2**: Individual attitudes toward business ethics affect one’s attitudes toward SR	Supported
**H3**: Intellectual openness positively affects individual SR attitudes	Rejected
**H4**: The larger the group size, the less likely it is for individuals to change their attitudes toward SR	Rejected
**H5**: The *strength of social relationship* among group members positively affects the change of ISR attitudes	Rejected
**H6**: Higher group engagement increases the likelihood that individual attitudes toward SR change as an effect of group activities	Supported
**H7a**: Individuals that highly value SR are more likely to stick to their initial opinion	Supported
**H7b**: Higher levels of group SR positively affect individual changes of attitudes toward SR	Supported

## Discussion

Acting as a ‘gap-spotter’ (Alvesson and Sandberg 2013), this study has addressed two existing issues in the literature. The first issue is that ethics and SR are usually considered (and measured) together with no clear distinctions being made between the two (Maignan 2001; Salmones et al. 2005; Singhapakdi et al. 1996). The second issue is that the literature has neglected socialization effects of the group on individual attitudes toward SR (Aguinis and Glavas 2012; Windsor 2001).

Acting as a ‘path-setter’ (Alvesson and Sandberg 2013) and theory builder (Colquitt and Zapata-Phelan 2007), this study provides some support for the fact that SR can be defined as *absolute,* which is a general, abstract tendency or idealistic view of business-society relations, and *relative*, an issue-based, specific view of SR, as it relates to a given problem, business, or case at hand. Furthermore, it presents a model to understand how these two layers of SR interact with group dynamics. This model has several key theoretical and managerial implications.

### Theoretical Implications

First, individual SR is not static. It changes via the socialization processes of group dynamics. In other words, when individuals interact with each other, their SR perception is likely to change. This finding suggests that SR should be addressed more from the social cognition perspective, as already suggested in a previous study (Secchi 2009) rather than from the more traditional approach that considers the individual in isolation (Aguinis and Glavas 2013).

Second, this study contributes to theory building (Colquitt and Zapata-Phelan 2007) by defining two layers of SR, and demonstrating how they change as a result of group interactions. The findings indicate that individual SR has two faces: one *relative* (specific to a given case or issue at hand), and one *absolute* (general propensity to view the nature of the relation between business and society universally). These two ‘faces’ are captured by *relative*-*SR* and *absolute*-*SR*; the two constructs affect and are affected by group activities differently. Thus, our findings suggest that individual perception of SR is multi-faceted. Most of the existing approaches and tools seem to lean toward the *relative*-*SR*, whereas we show that the phenomenon needs to be addressed more holistically. Groups are much diffused in everyday work life and many decisions are made through interactions (Levine and Moreland 1990). The point that emerges from our study, that first addresses the relative/absolute dimensions of SR for the first time, is that this distinction between the two SR should be taken into consideration.

Third, both *absolute*-*SR* and *relative*-*SR* impact attitudinal change (ACM) negatively. These are individual-level measures that change as a result of individual and group interactions. The attitudinal change assumes positive and negative values, depending on the direction of the change. For example, the negative coefficient suggests that the higher the initial SR, the lower the likelihood that it will change. This can be regarded as a *compensation effect*. Our findings show that the higher the group attitudes toward *relative*-*SR* or *absolute*-*SR*, the higher the impact on individual attitude change. On the one hand, participants with high SR are less likely to change. On the other hand, their high SR significantly affects other members of the group. From this result, a recommendation to organizations that work on the implementation of CSR is to build groups while keeping in mind what the average starting level of individual SR is among group members. One or more individuals scoring high (particularly in *relative*-*SR*, if we judge based on regression coefficients) is likely to affect the change of attitudes of the other group members.

Finally, the above points direct us to the role of groups in the shaping of SR thinking and, eventually, behavior (Ajzen 2005). As far as change of opinions is concerned, there are two concepts that can be considered. A group provides a good environment for people to consider or discount the opinions of others (Bonaccio and Dalal 2006; Moreland and Levine 2002). This study demonstrates that, contrary to previous findings in group research (Levine and Moreland 1990), the strength of social relationships (acquaintance), group size and, surprisingly, intellectual openness, may not be relevant for people to shape their ideas on SR. Instead, engagement in group activities seems to play a more significant role. A fundamental point here is that the more people feel engaged in and actively part of a group, the more their SR thinking is likely to change and adapt.

A group serves as a sort of cognitive *mediator* for its individual members (Magnani 2007). When individuals shape their cognition while *doing* (or, in our case, discussing) something, they are performing what Magnani (2009, p. 46) defines *manipulative abduction* (i.e., the idea of ‘thinking through doing’). The group is somehow ‘manipulated’ by individual members and facilitates cognitive processes that lead those individuals with higher SR to change and adapt more easily. In other words, this case could well serve as an example of how ISR works as socially distributed cognition (Alač and Hutchins 2004). In short, this opens an interesting subject for future research.

### Practical Implications

This study suggests practical implications in three areas of relevance—i.e., the nature of working relations, organizational structures, and training practices. Firstly, the literature on advice giving and taking suggests that expert advice has more chances to go through and be used by the decision maker (Sniezek et al. 2004). This could be a variable to take into consideration, although there is not enough information to derive this implication from the data presented here. The only information the data allow us to infer is that higher levels of ISR have a clear impact on groups and this may not necessarily mean expertise but it can be considered somehow similar to that.

On a different tack, findings highlight that there is no influence concerning the *strength of relations* among group members over the final change of attitude (ACM). This is particularly interesting when it comes to team building, contrary to what previous literature (e.g., Moreland et al. 1996) suggests.

The second practical implication concerns the impact of group work. Our research suggests that groups are a useful and effective means for conveying SR issues. Group work (slightly) moderates information on SR, so that organizations should think more seriously about group activities when implementing, discussing, and disseminating SR information to their employees. This is a particularly simple implication that may take the form of advice to managers: *use groups more often*. Although it is not clear what direction the group activities and individual attitudes will take, results show that change occurs through group dynamics. The time lag that we used for this study was minimal (1 week), but participants still showed significant change in both their relative and absolute attitudes toward SR.

Finally, this study was conducted among final year business students, who had at least one year of company work experience (the average is 3.25 years). These people represent the next generation of business managers, possibly with new perceptions and beliefs of SR in the current work environment. Therefore, what they think and perceive can be regarded as a marker for their future decisions as managers. Consistent with recent literature on samples (Shen et al. 2011), we argue that the use of these young people in this study is supported by the fact that we were exploring whether a phenomenon existed.

### Limitations

Although the study is ‘path-setting’ and was thoughtfully designed, there still remain some limitations that need to be solved in future research when the literature is more developed. The first concern relates to measures. We utilized those measures that seemed to be the most appropriate and relevant for our study. However, this is still an area where there is less research compared to that so far conducted on the organizational or corporate SR level of analysis. To ensure the appropriateness of the measures (reliability and validity), we ran several tests although further tests and analyses on other measures—for example, the multidimensional scale for ethical decision making (Casali 2011), the global social responsibility scale (Starrett 1996), or some other constructs from the so-called big five (Costa and McCrae 1992)—may offer additional ground to support (or reject) our hypotheses. Also, there may be more accurate measures to assess inter-personal relationships among group members.

Second, when we planned the quasi-experiment, we did not set the same group size for everyone. This might have allowed us to obtain more robust results, hence it may account for one of the limitations of this study. Moreover, there was no control group in the experiment (i.e., the control we used was size variation). This would have been particularly difficult since we could not justify excluding students from group work. This might be counted as another limitation. Future research will have venue for field study which might provide more factual findings than a quasi-experiment.

Finally, generalization of results may be hampered by the fact that the majority of participants came from the UK (87 %), though the sample size is a good fit for our analytical purposes (i.e., *N* = 461, statistical power is approximately .90). People from different cultures or from countries with different levels of economic development might perceive SR differently. Therefore, findings of this study should be interpreted with caution.

## Conclusion

This paper presented a study on how individuals change their attitudes toward SR as a result of group activities. We have isolated antecedents of ISR (i.e., business ethics and intellectual openness) and related them to group size, engagement, and strength of social relationships among members. The study shows that individuals have a different understanding of SR when they deal with a practical issue (*relative*-*SR*) as opposed to when they think of abstract business-society relations (*absolute*-*SR*). These two attitudes toward SR affect the way a group acts on individuals. Both *relative*-*SR* and *absolute*-*SR* negatively affect attitudinal change of opinion while the effect of the group engagement affects it positively. This opens venues for further theory development concerning how these two attitudes might affect organizational citizenship behavior, or decision making among business managers, for example.

The paper also suggests that SR is distinct from ethics, yet dependent on it; it is affected by, and significantly affects, group dynamics. Future studies should consider how individuals are facilitated in their attitudinal change when implementing, discussing, addressing, or assessing SR. This will stimulate scholars to reconsider and develop more theories on the interactions between individual and corporate social responsibility, the group being probably one of the many meso-levels of enquiry.

## Appendix 1

### Case: ReNu^®^. Too Much Cleansing!

Do you use contact lenses? Do you use them often? If your answer is ‘yes’ then there is a very good possibility that you know ReNu^®^, a solution for cleaning, disinfecting, and storing your contact lenses by Bausch and Lomb (B&L).

#### Bausch & Lomb: The Company

Founded in 1853, the company has its headquarter in Rochester (NY). It employs about 13,000 people and sells its products in more than 100 countries worldwide. The areas of operation are (a) contact lenses and related products, (b) pharmaceuticals, as well as (c) cataract and eye surgery.^1^

The company’s total sales for the year of 2005 were $2354 million, profits registered at $19.2 million; the revenue growth rate in the years 2003–2005 averaged 7.0 %. Their Research and Development (R&D) expenses totaled $177.5 million (7.5 % of revenues), and the operating income was stable at 12 % in 2005. The lens care segment of their business counts for about 18 % of their revenues.^2^ Their stocks are traded in the New York Stock Exchange.

In their 39-page Code of Business Conduct and Ethics, B&L state their purpose is to “stay true to a vision.” With a one-word/multiple-meaning statement, the vision is that of the founders: to improve the way people see. The Code states that the company “must maintain an ethical culture,” and they implement it with a commitment to ethical principles and with rules for sound decision making.^3^

#### ReNu^®^ with MoistureLoc

The solution, one of the company’s blockbuster products serves many purposes at the same time: it (a) cleans contact lenses and (b) removes proteins and other deposits that remain after use. The product accounted for $45 million of sales in 2004.^4^ The family of ReNu^®^ products are sold worldwide to an estimated 20 million customers.^5^

#### Some Troubling Results

It was February 20th, 2006 when the Health Ministry of Singapore issued a press release stating that out of 18 cases of serious eye infection, 100 % used B&L’s multi-purpose solution.^6^ The following day, the Health Ministry suggested that contact lens users stop using B&L’s ReNu^®^ “because of a ‘very strong association’ between the solution and a recent spate of fungal corneal infections.”^7^ The number of people affected at that time in Singapore was 39, of which 34 said they used the solution. According to the Malaysian New Straits Times^8^ similar cases were found in Hong Kong. Far from being isolated to the Asian market, similar cases were found in the United States. The eye infection was found in 109 contact lens users over a 10-month period ending April 2006. The U.S. Centers for Disease Control and Prevention reported that the infection was *fusarium keratitis*, a corneal infection that, in some cases, may result in serious corneal injuries and blindness if a transplant is not performed.^9^

The Food and Drug Administration (FDA) decided to inspect B&L’s manufacturing facility, located in Greensville, S.C.^10^ The inspection was supposed to determine the fate of the case.

According to Ron Zarrella, the company’s CEO, the solution was safe. On April 12, he claimed, “tests had shown the lens cleanser ReNu with MoistureLoc was effective in killing the *fusarium keratitis* fungus that causes the infection on the cornea.” Moreover, he also stated that ReNu^®^ is “as safe and effective as anything on the market” and that “there’s no indication there is a formula problem here.”^11^

Market analysts warned of dangers “that there is permanent damage to the brand”^12^ and the share price started plummeting as soon as the news of the infection was released in the U.S.^13^ In the meantime, major drugstore chains in the U.S., including CVS, Walgreens, and Jewel-Osco, started to wonder whether to take actions to protect their customers instead of awaiting the company’s decision.

#### Open-Ended Questions (Not Used in the Analyses)

1. What are the alternatives that the company faces? And what are the consequences related to each alternative? Try to answer as if you were a manager of the company.
2. How many dimensions/types of social responsibility can be related to the case?
3. On the basis of your answer to question 2, what is the strategy that you think managers should embrace to effectively deal with the situation?

## Appendix 2

With the aim of contributing to the diffusion of research in individual and group social responsibility, Appendix 2 includes all the measurement variables used in this study. The two key variables (*absolute* and *relative* SR) are presented with a higher degree of detail.

### Ethical Judgement

[7-point bipolar scale]

**Table:**

Unfair	1	2	3	4	5	6	7	Fair
Unjust	1	2	3	4	5	6	7	Just
Unacceptable to my family	1	2	3	4	5	6	7	Acceptable to my family
Not morally right	1	2	3	4	5	6	7	Morally right
Culturally unacceptable	1	2	3	4	5	6	7	Culturally acceptable
Traditionally unacceptable	1	2	3	4	5	6	7	Traditionally acceptable
Violate unspoken promise	1	2	3	4	5	6	7	Not violate unspoken promise
Violate unwritten contract	1	2	3	4	5	6	7	Not violate unwritten contract

Adapted from Reidenbach and Robin (1990)

### Relative-SR

[1 = strongly disagree; 7 = strongly agree]

The measure for *relative social responsibility* was revised from Garcia de los Salmones et al. (2005). We adjusted the items to fit the case presented to participants but the measure is very much the same as the original:

*State whether you disagree or agree with the following statements about the company (B&L)*

1. tries to obtain maximum profit from its activities
2. tries to obtain maximum long-term success
3. always tries to improve its economic performance
4. always respects the norms defined in the law when carrying out its activities
5. is concerned to fulfill its obligations vis-a`-vis its shareholders, suppliers, distributors, and other agents with whom it deals
6. behaves ethically/honestly with its customers
7. respects ethical principles in its relationships
8. is concerned to respect and protect the natural environment
9. is concerned to improve the general well-being of society

### Absolute-SR

[1 = non-existent/irrelevant; 7 = core value/totally relevant]

The measure for absolute social responsibility was developed following a two-step procedure. First, we listed 28 expressions that we thought could represent what a socially responsible business should do. Second, we submitted the list to colleagues with expertise in the field of either ethics, social responsibility, and scale development (DeVellis 2012) and asked to assess whether these expressions represented *absolute-SR* (spelling out its meaning). After a few rounds we then retained 12 items. Third, we pilot tested the measure with a small random sample of participants and ran preliminary EFA and CFA. We repeated these tests for measure validation and performed an EFA with participants of the first wave (*n* = 276); results are the following:

**Table:**

1. Proactive business strategy	0.636
2. Corporate values	0.752
3. Employees rights	0.743
4. Economic performance	0.505
5. Societal care	0.708
6. Philanthropy	0.632
7. Diversity promotion	0.636
8. Law-abiding behavior	0.756
9. Ecological responsibility	0.683
10. Product quality	0.706
11. Risk seeking	0.300
12. Human rights	0.778
Total variance explained	0.443

Item 11 loads poorly on the scale compared to the others (<0.40). A subsequent CFA performed with the data from the post-test for participants of both waves confirms that a measure with 12 items loads well on one factor: *χ* ^2^ = 112.511 [*df* = 35], *p* < .001; *χ* ^2^/*df* = 3.215; CFI = .966; RMSEA = .069; SRMR = .041; BIC = 15883.809, although the *χ* ^2^/*df* is not within the recommended range. If we consider a 11-item measure, results are slightly better: *χ* ^2^ = 73.085 [*df* = 27], *p* < .001; *χ* ^2^/*df* = 2.706; CFI = .979; RMSEA = .060; SRMR = .036; BIC = 14376.349. The difference between the aggregated 11-item and 12-item scale was not statistically significant; hence, we kept the 12-item scale leaving more fine-grained discussions of validity/reliability to a measurement paper. The test we performed and reported in the paper are conducted with the larger sample, this is why some of the indices report slightly different (still consistent) values. The scale construction procedure we followed together with these preliminary tests indicates some support for the validity of the proposed scale. Full validation and reliability analysis will be published in a separate paper, including a discussion on whether this scale can be considered a reflective (as we did here) or a formative scale. The way values are aggregated do not pose threats to the measure in the analysis of this paper.

As a final step in our process, we included the scale in the quasi-experiment. Here are items and wording that we used for the scale of *absolute*-*SR.*

*Describe what you think for each of the following words and expressions as core values of operating businesses. Use numbers from 1 to 7, where 1 is “non-existent/irrelevant” and 7 is “core value/totally relevant.*”

**Table:**

Proactive business strategy	1	2	3	4	5	6	7
Corporate values	1	2	3	4	5	6	7
Employees rights	1	2	3	4	5	6	7
Economic performance	1	2	3	4	5	6	7
Societal care	1	2	3	4	5	6	7
Philanthropy	1	2	3	4	5	6	7
Diversity promotion	1	2	3	4	5	6	7
Law-abiding behavior	1	2	3	4	5	6	7
Ecological responsibility	1	2	3	4	5	6	7
Product quality	1	2	3	4	5	6	7
Risk seeking	1	2	3	4	5	6	7
Human rights	1	2	3	4	5	6	7

### Intellectual Openness

[1 = strongly disagree; 7 = strongly agree]

**Table:**

1. I carry the conversation to a higher level
2. I prefer to stick with things that I know
3. I am interested in many things
4. I prefer variety to routine
5. I am not interested in abstract ideas
6. I am not interested in theoretical discussions
7. I want to increase my knowledge
8. I rarely look for a deeper meaning in things
9. I am open to change
10. I try to avoid complex people. From Jackson et al. (2000)

### Group Engagement

[1 = strongly disagree; 7 = strongly agree]

*During the discussion I believe that…*

1. I felt comfortable expressing my opinions
2. I have always been keen on accepting opinions from other members of the group
3. Overall, the opinion of the group was very similar to what I was thinking before.

## Abbreviations

ACM: Attitude change measurement
AGSR: SR After group activities
ANOVA: Analysis of variance
BGSR: SR Before group activities
BIC: Bayesian information criterion
CFA: Confirmatory factor analysis
CFI: Comparative fit index
CSR: Corporate social responsibility
ICC: Inter-correlation coefficient
ISR: Individual social responsibility
MRCM: Multilevel Random Coefficient Model
OLS: Ordinary least squares
RMSEA: Root mean square error of approximation
SR: Social responsibility
SRMR: Standardized root mean square residual
WOGA: Weight of group activities
